# Whole exome sequencing reveals rare variants linked to congenital pouch colon

**DOI:** 10.1038/s41598-018-24967-y

**Published:** 2018-04-27

**Authors:** Praveen Mathur, Krishna Mohan Medicherla, Spandan Chaudhary, Mruduka Patel, Prashanth Bagali, Prashanth Suravajhala

**Affiliations:** 1 Department of Pediatric Surgery, SMS Medical College and Hospital, JLN Marg, Jaipur, 302004 RJ India; 2 Department of Biotechnology and Bioinformatics, Birla Institute of Scientific Research, Jaipur, 302001 RJ India; 3 Division of Genomics, Bioinformatics and Diagnostics, Xcelris Labs Limited, Ahmedabad, India

## Abstract

We demonstrate the application of whole exome sequencing to discover the rare variants for congenital pouch colon, acronymed CPC. For 18 affected individuals in a total of 64 samples, we sequenced coding regions to a mean coverage of 100×. A sufficient depth of ca. 94% of targeted exomes was achieved. Filtering against the public SNP/variant repositories, we identified a host of candidate genes, EPB41L4A and CTC1 associated with colon, neural/brain muscles and Dyskeratosis Congenita maladies. Furthermore, the stop gain mutations in the form of JAG1,OR5AR1,SLC22A24,PEX16,TSPAN32,TAF1B,MAP2K3 and SLC25A19 appears to be localized to Chromosomes 2, 11, 17 and 20 in addition to the three stop lost mutations across three genes, viz. OAS2, GBA3 and PKD1L2 affecting the colon tissue. While our results have paved way for transcendence of monogenic traits in identifying the genes underlying rare genetic disorders, it will provide helpful clues for further investigating genetic factors associated with anorectal anomalies, particularly CPC.

## Introduction

Congenital Pouch Colon (CPC) is a congenital malformation in which the entire large bowel or its segments of varying lengths exhibit enormous dilatations in the form of a pouch and communicate distally through a fistula with urogenital system^1–3^. The largest patient series in CPC are being reported exclusively from India. The incidence of CPC is the highest in the North West regions of India and is estimated to be 5–18% of the total number of neonates managed for anorectal malformations. The CPC more frequently affects the male population with a male:female distribution of 4:1^4^. Plain erect abdominal radiographs and conventional invertogram are sufficient to establish the diagnosis of CPC. Based on the anatomic morphology, the CPC is classified into five types^5^. Surgical management depends on the type of CPC, and outcomes are variable depending on the length of the colon affected and anorectal muscle complex. Since 2005 and aftermath of the human genome project, several efforts have been made to understand the clinical genetic makeup of CPC^5,6^. However, no studies at the sequence level have been reported where the genetic variants have been shown to be associated with CPC. In this study, we have reported genetic variants associated to CPC of 18 cases in northern India. We performed whole exome sequencing (WES) for a total of 64 samples including the unaffected siblings/parents, where available. The samples were analysed for mutations predicted to be causal. The datasets were filtered against GeneMania reference and checked for significant association of deleterious heterozygous germline mutations. While a marked impact on the risk of CPC specific genes in affected individuals versus controls was studied using WES, we claim that this is the first study of its kind where exomes were analysed for genetic evaluation of CPC. With the 1000 genome project advancing to a next level, genetic makeup for such rare diseases could be characterized better^7^. The downstream analysis containing an overview of the genetic interactions and pathways associated with CPC related genes is discussed in detail.

## Results and Discussions

### Characteristics of CPC

**Figure 1:**
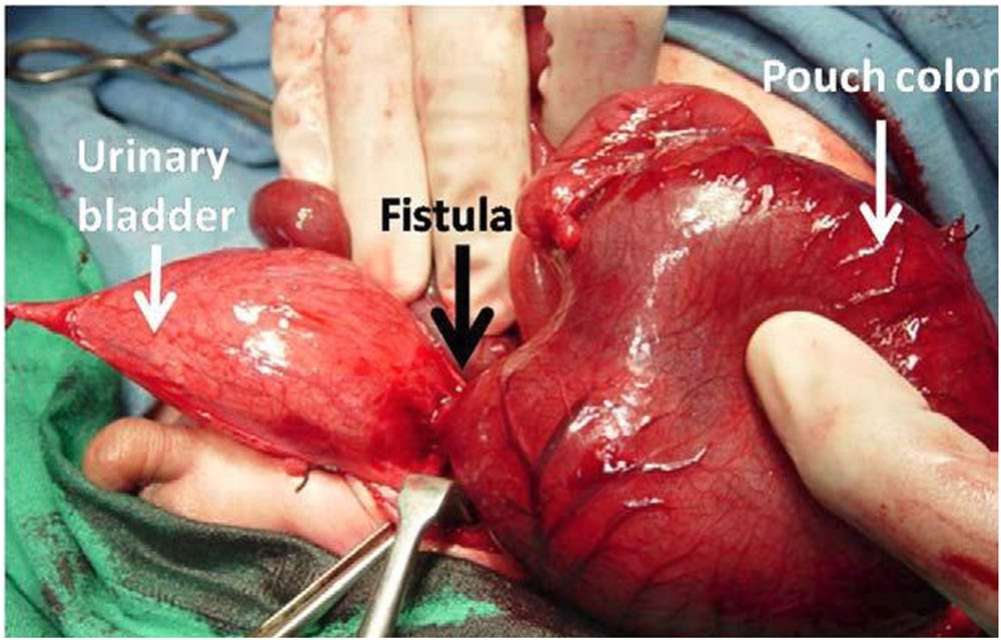
Clinical characteristics of CPC. Image courtesy of Mathur *et al*., (2008).

The standard management of CPC is done by staged surgical procedures employing the placement of protective stoma (Ileostomy or Colostomy) with ligation of fistula in the first stage followed by a definitive pull-through in the second stage. Delayed presentations are commonly associated with colonic pouch perforation leading to peritonitis and septicemia that are largely responsible for high mortality (see Fig. 1).

### Whole exome sequencing

A total number of 64 subjects including 18 affected neonates along with their parents and unaffected siblings were subjected to WES, which was followed by validation of selected genes by dideoxynucleotide sequencing (Sanger sequencing) method. We found that all the samples passed the sequencing quality thresholds. The sequences were then aligned to human genome reference (hg38) using bowtie2^8^ to produce the sequence alignment file. The contamination crosscheck using verifyBAMID^9^ was done to assess the heterogeneity of each given site of pooled dataset. All the individuals reported less than 2% contamination with optimal per sample f-mix of 0.01 to further allow segregation analysis and inheritance patterns of genes in affected individuals. Variant analysis using Vt and Annovar predicted the variant types and were filtered by setting criteria for false discovery rate (FDR). We observed a total 14,12,1242 (14.12 million) annotated variants and upon further normalization, we discovered 8601 variants, among which, 7949 were biallelic indels and 652 were multiallelic indels. The false positives in this process were carefully inferred for understanding exomes of healthy individuals *versus* affected individuals. Where results were clear, a number of exome sites, low-coverage sites and large deletions could not be validated (Supplementary Table 1). The initial indel call yielded average depth coverage of 33.9264 and 40.9896 for Vt and Varscan respectively. We observed 63484 heterozygous variants which confirm that the variant calls with above thresholds were filtered across all samples. In addition, the low-coverage SNPs and indel sites were found to be variable which is in concordance with characterizing variation to low-complexity genomic regions and the amount of other data pertaining to the fraction of the reference genome leading to reliable variant discovery. The common variation associated with CPC across coding consequences revealed that 8158 genes overlapped across 38566 transcripts yielding 7043 variants (Supplementary Table 1).

### Association of rare variants with CPC

**Figure 2:**
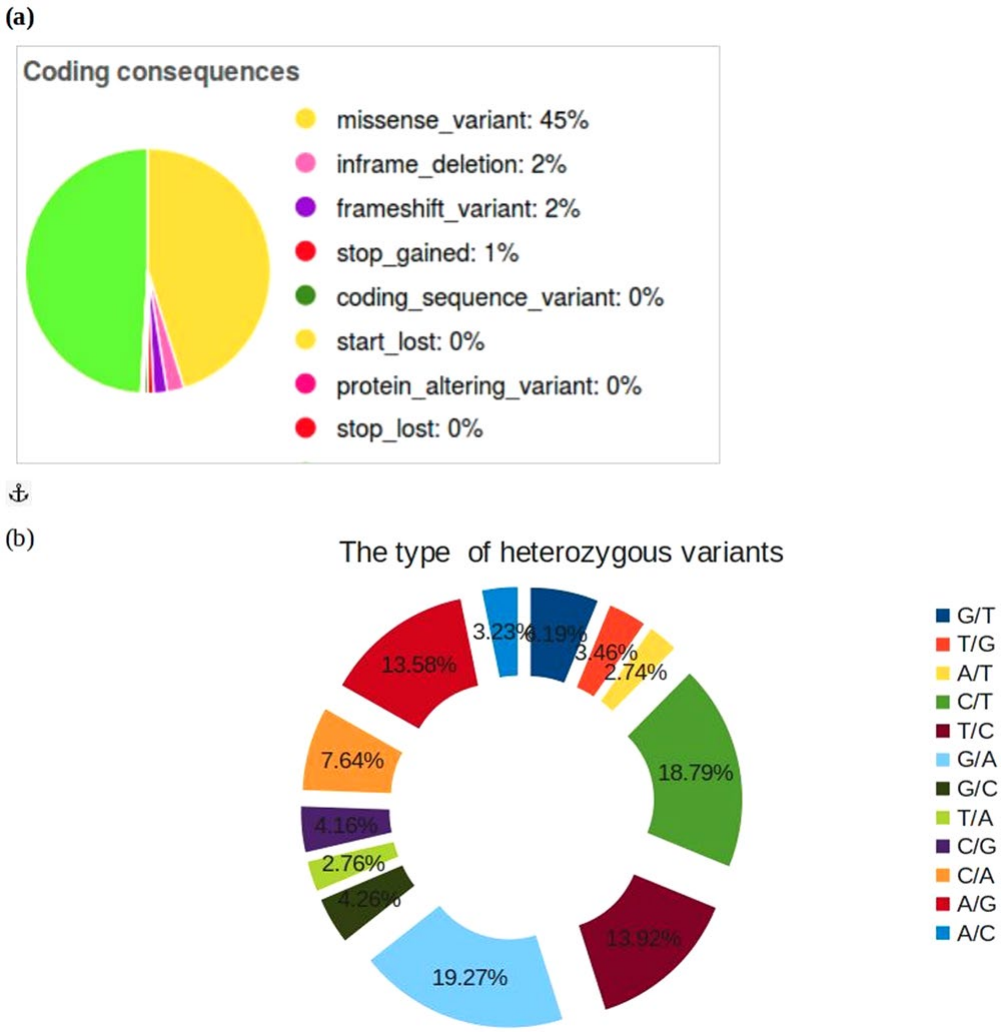
Number of candidate variants in the form of (**a**) coding sequences and (**b**) “heterozygous” mutations filtered against variation databases and ensembl suites.

The germline variants often tend to be false positives and are rare mutations. Assuming every newborn has ca. 70 *de novo* variants^10^, detecting phenotype’s rarest events could not be achieved with this power. Hence, to rule out the false positives, all SNPs from 1000 genomes were downloaded and mapped to the variant effect predictor (VEP) ensembl suite^11^ to detect the RefSeq (rs) ids for all the SNPs that have minor allele frequency cutoff 0.05 and those that intersect the data matching the allele frequency (Fig. 2). With the growing debate over the genetic contribution associated with complexity of diseases, consistent low penetrant variants tend to be purifying form in a large amount of disease susceptible genes which is in agreement with the ‘Common Disease, Rare Variant (CDRV)^12^.

### A two fold increase in burden of conserved mutations in affected vs. unaffected individuals

We expected to have a major difference in functional alleles when compared to unaffected controls as evident from the analysis. Apart from exploring the singleton mutations, we used a variety of criteria to distinguish the subset. To our knowledge, there have been no studies of exomes or rare variant mutation burden in these genes or exome data. Since there is a correlation between the number of synonymous mutations with the numbers of rare (MAF cutoff 0.05%), we observed the impact of rare missense variant. Of 1064 missense variants, we detected 24 distinct missense variants that are associated with several unannotated hypothetical genes/open reading frames. There are two such variants mapped across the same gene (C10orf53, C10orf120 and C7orf31 and c7orf57) while all of them map to 11 different chromosomes (Supplementary Table 2). As the missense variants are located throughout these ORFs, potential functional implications associated with them at the non-coding or intron level cannot be ruled out^13^.

### Frameshift mutations, stop-gain as high impact variants for CPC

**Figure 3:**
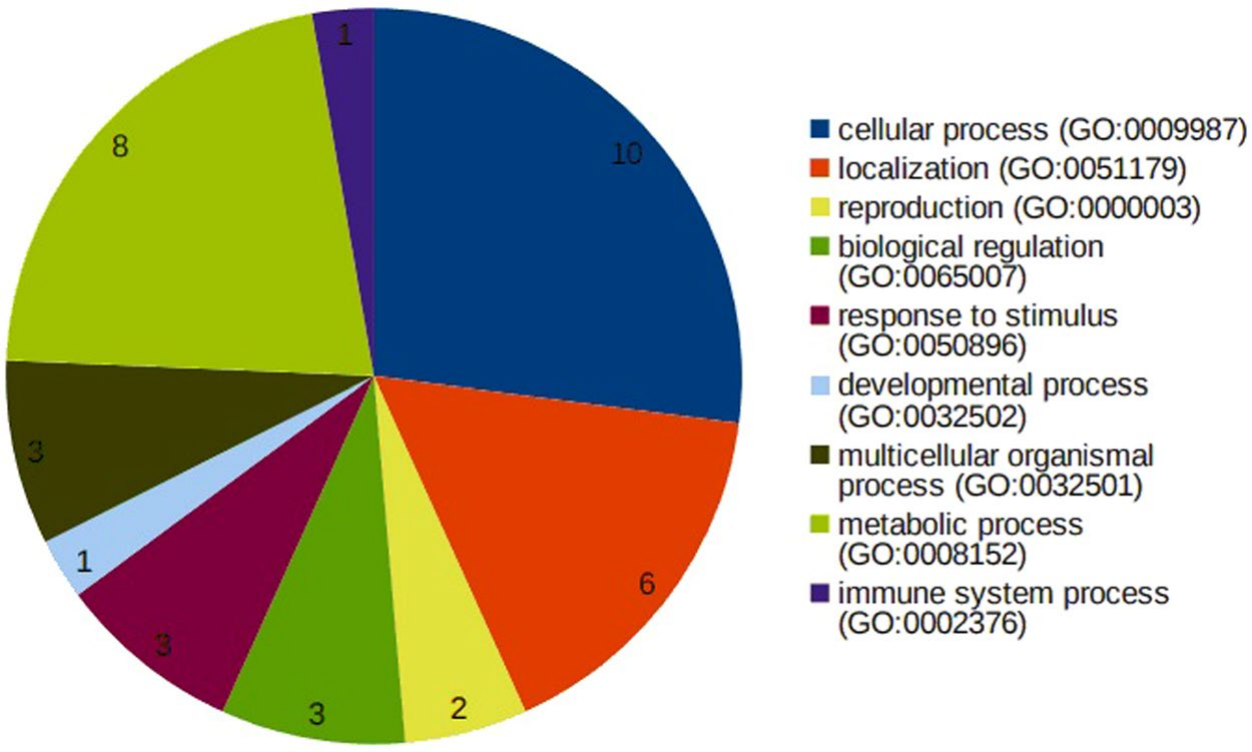
Frameshift variants associated with various GO processes.

Among the variants observed are frameshift, loss or gain of stop codons which have significant insertions/deletions at a number of bases in different reading frames with altered protein sequences. Two of the 63 frameshift variants affected with spliced regions are EPB41L4A and CTC1 genes associated with colon, neural/brain muscles and Dyskeratosis Congenita maladies (Supplementary Table 3) and when mapped to the gene ontology (GO) pathways, they are widely distributed (Fig. 3). Considering the fact these splice site variants may influence tissue progression, there is a high chance that these gene mutations might be associated with malformation of the colon tissue. The stop gain mutations were observed in nine genes, viz. JAG1, OR5AR1, SLC22A24, PEX16, TSPAN32, TAF1B, MAP2K3 and SLC25A19 which are localized to chromosomes 2, 11, 17 and 20. We also observed three stop-lost mutations across three genes, viz. OAS2, GBA3 and PKD1L2 affecting the colon tissue localized to chromosomes 12, 4 and 16, respectively (Supplementary Table 4a).

### Gene enrichment and pathway analyses

**Figure 4:**
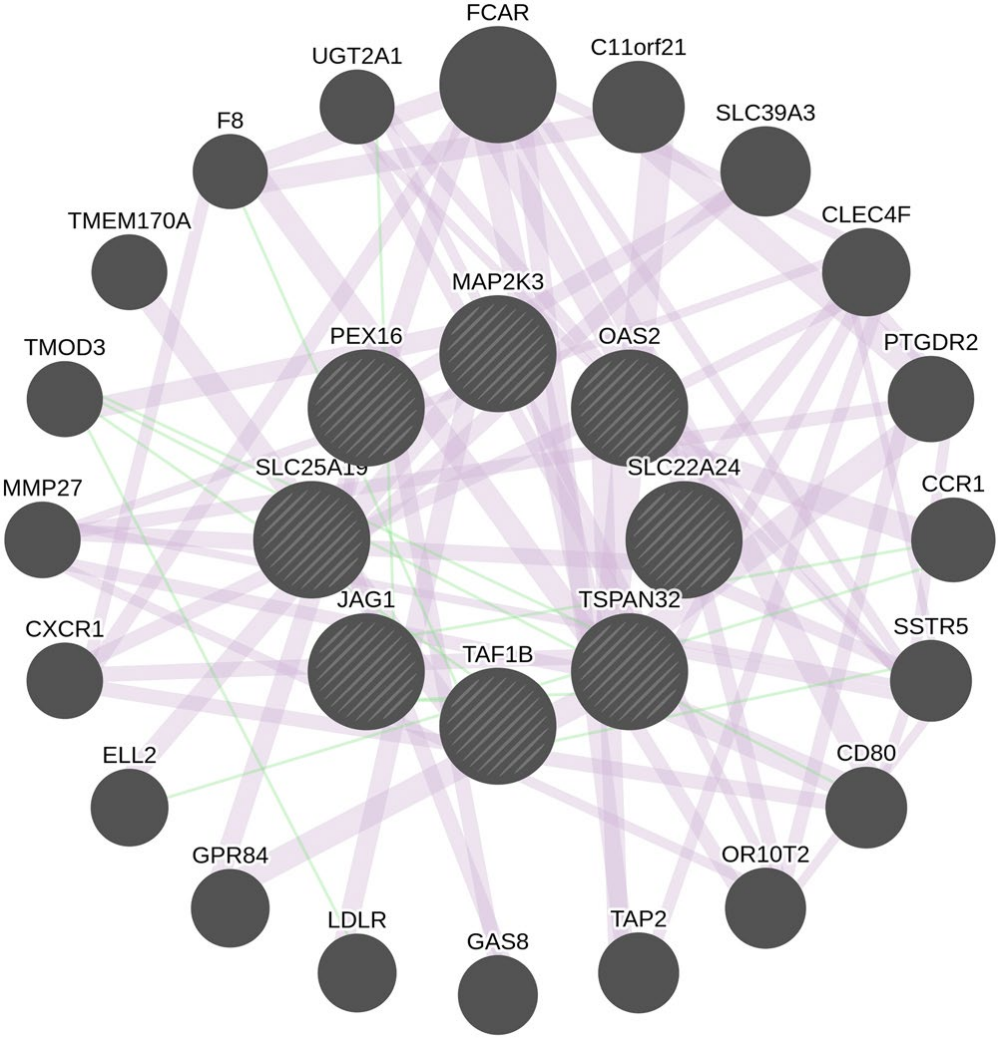
A network of peer associations with CPC causal genes. The central nodes with blue edges form the coexpressed networks.

When these 11 genes were subjected to genemania, a large number of them were shown to be coexpressed (99.67%) while a small set (0.33%) makeup the genetic associations (Fig. 4). This shows that majority of them are associated with lectin mediated colon interactions. To test an excess of rare variants across all genotypes, the genes inferred from genemania predictions were integrated with biological associations.

### Sanger validation of CPC variants

Sanger validation (orthogonal) was used to incorrectly disprove a true-positive variant. We performed a systematic validation of variants from 14 genes using Sanger sequencing across all affected individuals and found no discrepancies among them (Supplementary Table 6a and b).

### Population stratification at a gross-level yielded the state of art of rare genetic disorder

**Figure 5:**
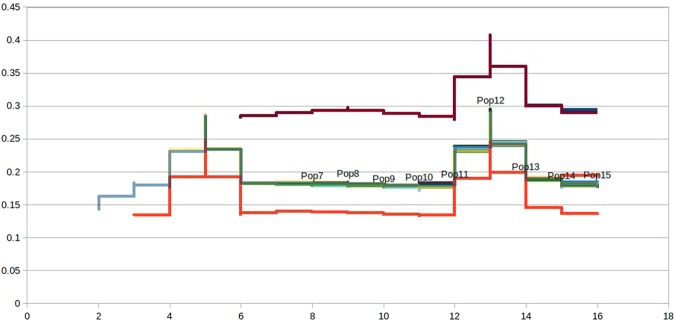
Population strata of CPC infected samples indicating coherence (green and purple lines). The x axis represents the 18 affected samples with y axis showing effective MAF 0.5.

We reviewed the patterns of stratified population and asked if they were influenced to vary between common and rare variants. In principle, the rare variants were found to have stronger patterns when compared to the common variants. Assessing these variants in CPC has provided an ample evidence of strata coherent to CPC traits (Fig. 5).

## Materials and Methods

### Patients and samples

The CPC subjects included in this study were all selected from the SMS hospital, Jaipur registries during 2011–’16. A written informed consent was obtained from all subjects sampled. All patients that were invited to participate had consent/ethical concerns achieved from their parents and unaffected older siblings. The parents had disease-free-match as CPC controls. Parents and unaffected siblings were asked to donate a 2 ml blood sample while from neonates; peripheral vein sampling for exome sequencing was done. This study received approval of the ethical committee of SMS hospital and Indian Council of Medical Research (ICMR), New Delhi. All methods were performed in accordance with the relevant guidelines and regulations. A total 64 samples including 18 affected neonates along with their parents and unaffected siblings have been sequenced. All raw/throughput data are performed in-house using our power Dell machine with 64GB RAM.

### Exome capture and sequencing

Exome sequencing was carried out at the Xcelris Labs using genomic DNA (gDNA) extracted from clinical tissue samples using QIAamp® DNA Blood Mini Kit (Cat No :51104) followed by quantification and quality check of the gDNA using Qubit® 2.0 Fluorometer and agarose gel electrophoresis respectively. Around 200 ng of gDNA was used for fragmentation using Covaris S2 system to generate fragments with average size from 150 bp to 200 bp followed by endrepair, adapter ligation and amplification. Amplified adapter ligated DNA was subjected to hybridization for capturing exonic regions using provided RNA baits (V5 + UTRs). Exome capture was performed using SureSelectXT Target Enrichment System for Illumina PE Multiplexed Sequencing Library as per manufacturer’s instructions. Agilent SureSelect All Exome Kit (Mb) and the Human All Exon 75 Mb kit covering exons, intron exon boundaries and UTR regions captured targeted exonic regions amplified to generate final library which was followed by quality check using high sensitivity (HS) chip on Agilent bioanalyzer 2100 (Agilent Technologies). Size of all the libraries were ranging from 321 bp to 423 bp. Library quantification was done using quantitative PCR (qPCR) before sequencing run (*see* Supplementary Table 7). All the libraries were sequenced on Illumina NGS platform to generate pair end reads with 100× depth coverage which resulted into 8–10 gb of data per sample.

### Quality control (QC) and variant calling

**Figure 6:**
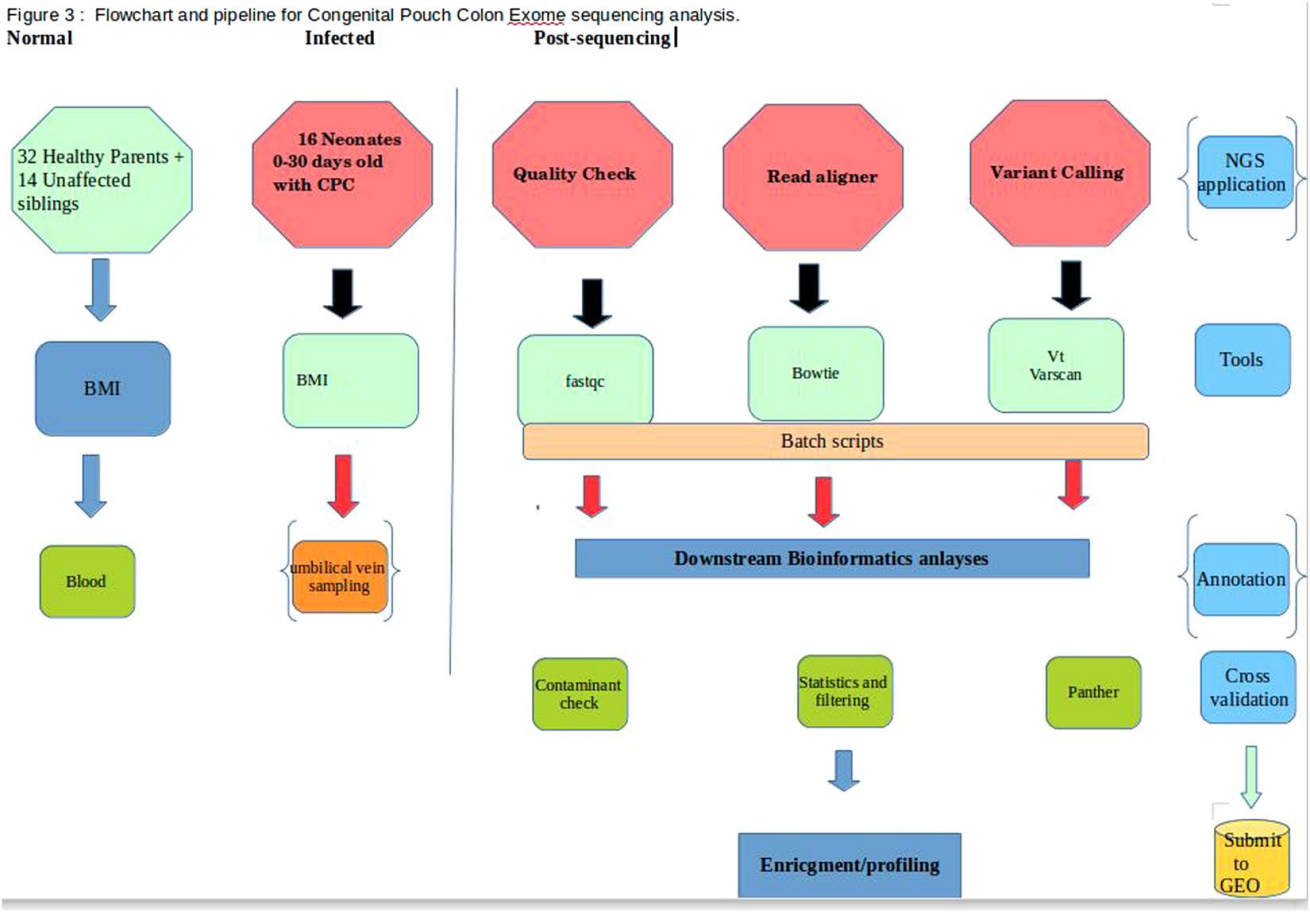
An in-house pipeline used after the initial quality checking which aligns the reads and calls the variants before cross-validating and downstream analyses (Supplementary Information).

The quality assessment was done for all the samples using FastQC^14^ with raw reads checked for quality, GC bias, K-mer quality, duplication levels. Calling and filtering of variants and indels was done by a wide number of tools, *viz*. Varscan^15^, Annovar^16^ and vt^17^ which compares known sites in variant databases with parameters set (Fig. 6) to establish sensitivity and specificity of variants calling. Mutations were counted as heterozygous (“het”) using awk/bash one-liners. Further predictions identifying “deleterious” mutations were screened for sanger validation.

### Sanger sequencing and validation of SNPs

**Figure 7:**
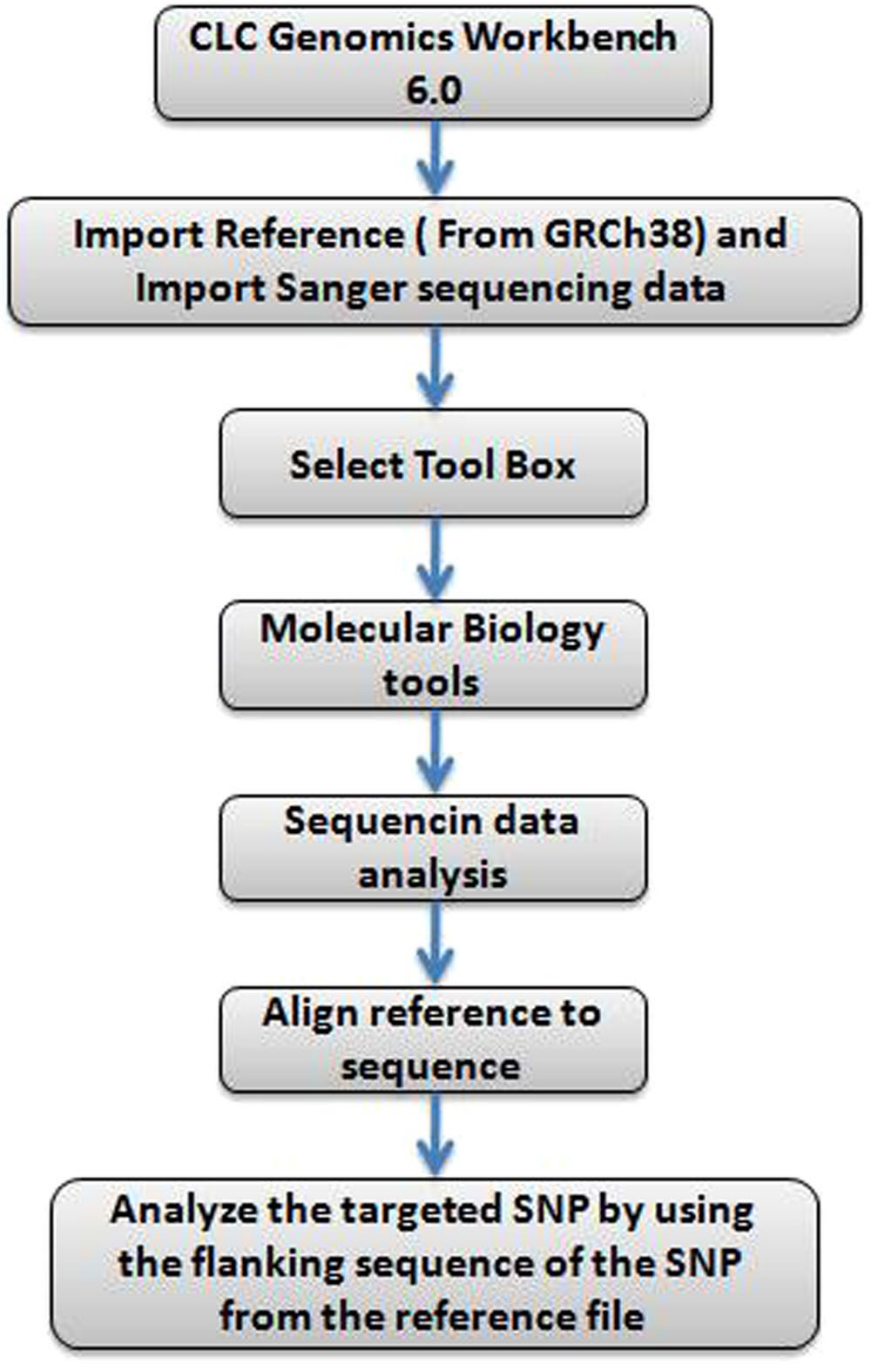
SNP validation workflow using CLC genomics workbench.

Based on the whole exome sequence data analysis of 18 probands and 14 siblings, we have chosen a set of 14 SNPs that exhibited significant association with CPC in affected subjects. Fourteen SNPs were subjected to further validation in an independent platform, namely dideoxynucleotide sequencing using ABI3730xl. PCR primers for covering 14 mutations or SNP position were designed manually with the help of freely available tool “OligoCalc (Oligonucleotide Properties Calculator)” at Xcelris labs (Supplementary Table 5). Primers were designed with a length of 18–25 nucleotides. For ease of handling and standardization of primer, universal M13 primer sequences were fused with our designed primers. We have maintained an average of 400–600 bp of product size with the 200–250 bp flanking from both sides of the location of SNP mutation. Primers were designed in such a way that secondary structures in genome are avoided. We adjusted GC content as 50% to maintain a balanced distribution of GC-rich in CDS regions. All the 32 individual samples (DNA samples) were amplified using designed primers. PCR condition used was initial denaturation at 95 °C for 5 minutes followed by 35 cycles of denaturation at 95 °C for 30 seconds, annealing and extension at 72 °C for 45 seconds and final extension of 5 minutes at 72 °C. Annealing temperature used for *KIF13A* gene was 56 °C and 60 °C for *PTK7, E2F6, BAIAP3, IL33, EGFLAM, Clorf105, PAN2* and 62 °C for *BTN1A1, ZNF346* genes. All the amplicons were of good quality. The PCR amplicons were purified with ExoSAP (USB) or bead based purification (AMPure Agencourt XP beads) and subjected to automated DNA sequencing on ABI 3730xl Genetic Analyzer (Applied Biosystems, USA). Sequencing was carried out using Big Dye Terminator v3.1 Cycle sequencing kit following the manufacturer’s protocol, where sequencing cycle was set with the thermal ramp rate of 1 °C per second for 30 cycles. Sequence data of 32 samples for targeted 14 SNPs were analyzed using CLC workbench tool using the human reference genome assembly GRCh38 (refer Fig. 7) keeping approximately 500 bp sequence flanking of targeted SNPs (given in Supplementary Table 6a).

### Statistical analyses and population stratification

All statistical tests were done as elusive. Sample relationship checks were done using the Plink and we obtained the pedigree (.ped) and family (.fam) files. The obtained files were further used to filter the variants using Plink and binary (.bin) and bed graphic format (.bed) were retrieved with a MAF of 0.05 which were then used for mapping them VEP. Principal components analysis for population stratification was performed with the data from all the merged samples. The 1000 genomes dataset containing the datasets was considered as a summarized worldwide population. In order to estimate individual ancestries, we used an unsupervised ADMIXTURE^18^ (Alexander *et al*. 2009) to project the samples.

### Contamination Check

VerifyBamID was used to infer whether or not the reads are contaminated across the sample swaps. All the 18 affected cases specifically were checked for robust detection of sample and per-sample freemix was calculated wherein the depth distribution was used for performing with-chip estimation of sample mixture at fixed reference.

### Downstream bioinformatics analyses

To integrate the results with different pathways, we used “gene-level” annotation using PANTHER^19^ which assesses the ontology/pathway effect of the “mutated” gene across assorted databases like clinvar, dbSNP. The variation in coding sequences resulting in potential changes in protein sequences when compared to the variation observed in non-coding regions as a neutral was detected. In addition, global enrichment analysis and association networks were inferred using GeneMania^20^ to create and visualize gene networks by evidence in pathways and protein-protein interactions (predicted and experimental).

### Portions of variants called for candidate prioritization that is sensitive

There are several tranches that are sensitive and therefore variants that are frame shift, stop gained and stop lost subtly are linked to “rare” events. While we looked at the variants of each individuals sequenced, hard filtering on parameters and duplicated variants were carefully removed. List of SNPs that remain after all filtering the duplicates across all high frequency, SNPs and variants were used as a final dataset to consider them as probably causal. After we set the threshold MAF to >0.5% and prioritized the variants, we went on to segregate the variants across the infected and compared them against the unaffected siblings. A careful search on interesting variants in the affected and the either parents each carrying allele were used to screen pathogenic variants as per co-segregation rules.

## Conclusions

In this study, we performed the whole exome sequencing of CPC samples and confirmed significant associations in diseased subjects with rare mutations and variants. Further analyses revealed identification of variants related to urogenital diseases. Estimating the age of rare genetic variants can be a confounding factor to decipher connections between populations^21^. Exploring the state of origin of nonfunctional variants will help us understand the incidence of the disease in north western India. However, these preliminary findings should be augmented by further analyses using a larger cohort of trio exomes. With the goal set for easy identification of CPC using NGS technologies, a panel database including tested genes, diseased phenotype and OMIM panel numbers for such rare genetic disease could be of significant interest to clinical NGS community.

## Data Availability

The data analysed in house is available for reference.

## Electronic supplementary material

Supplementary_Table 1

Supplementary_Table 2

Supplementary_Table 3

Supplementary_Table 4a

Supplementary_Table 4b

Supplementary_Table 5

Supplementary_Table 6a

Supplementary_Table 6b

Pipeline used for WES analyses
