# Author Correction: Whole exome sequencing reveals rare variants linked to congenital pouch colon

**DOI:** 10.1038/s41598-018-32119-5

**Published:** 2018-09-11

**Authors:** Praveen Mathur, Krishna Mohan Medicherla, Spandan Chaudhary, Mruduka Patel, Prashanth Bagali, Prashanth Suravajhala

**Affiliations:** 1 Department of Pediatric Surgery, SMS Medical College and Hospital, JLN Marg, Jaipur, 302004 RJ India; 2 Department of Biotechnology and Bioinformatics, Birla Institute of Scientific Research, Jaipur, 302001 RJ India; 3 Division of Genomics, Bioinformatics and Diagnostics, Xcelris Labs Limited, Ahmedabad, India

Correction to: *Scientific Reports* 10.1038/s41598-018-24967-y, published online 27 April 2018

The original version of this Article contained errors in the References. The following was erroneously included as Reference 7, and has now been removed:

Pandey, V., Gangopadhyay, A. N., Gupta, D. K. & Sharma, S. P. Single stage management of a unique variant of congenital pouch colon with triplet fistula and normal anus. *J Indian Assoc Pediatr Surg*. **20**(3), 148–9 (2015).

Additionally, the original version of Article contained typographical errors in the Abstract and Conclusions.

In the Abstract:

“Furthermore, the stop gain mutations in the form of JAG1,OR5AR1,SLC22A24,PEX16,TSPAN32,TAF1B,MAP2K3 and SLC25A19 appears to be localized to Chromosomes 2, 11, 17 and 20 in addition to the three stop lost mutants across three genes, *viz*. OAS2, GBA3 and PKD1L2 affecting the colon tissue.”

now reads:

“Furthermore, the stop gain mutations in the form of JAG1,OR5AR1,SLC22A24,PEX16,TSPAN32,TAF1B,MAP2K3 and SLC25A19 appears to be localized to Chromosomes 2, 11, 17 and 20 in addition to the three stop lost mutations across three genes, *viz*. OAS2, GBA3 and PKD1L2 affecting the colon tissue.”

In the Conclusions:

“With the goal set for easy identification of CPC using NGS technologies, a panel database including tested genes, diseased phenotype and OMIM panel numbers for such rare genetic disease could be of significant interest to clinical NGS community^22^.”

now reads:

“With the goal set for easy identification of CPC using NGS technologies, a panel database including tested genes, diseased phenotype and OMIM panel numbers for such rare genetic disease could be of significant interest to clinical NGS community.”

These errors have now been corrected in the PDF and HTML versions of the Article.

