# Supplementary material for: Whole exome sequencing reveals rare variants linked to congenital pouch colon
Source: Sci Rep. 2018 Apr 27;8:6646. doi: 10.1038/s41598-018-24967-y (PMC5923232; doi:10.1038/s41598-018-24967-y)
Supplement: Supplementary file 9 — Pipeline used for WES analyses [file 41598_2018_24967_MOESM9_ESM.docx]

| **Supplementary Information**  **Whole Exome Sequencing reveals rare variants linked to Congenital Pouch Colon**  Praveen Mathur^1^, Krishna Mohan Medicherla^2^, Spandan Chaudhary^3^, Mruduka Patel,^3^ Prashanth Bagali^3^, and Prashanth Suravajhala^2*^  1. Department of Pediatric Surgery, SMS Medical College and Hospital, JLN Marg, Jaipur 302004 RJ, India  2. Department of Biotechnology and Bioinformatics, Birla Institute of Scientific Research, Jaipur 302001 RJ, India  3. Division of Genomics, Bioinformatics and Diagnostics, Xcelris Labs Limited, Ahmedabad, India  *Correspondence: [prash@bisr.res.in](mailto:prash@bisr.res.in)  #Indexing already done using bowtie2, BWA and samtools: ../data/hg38/ |
| --- |
| #All scripts and commands are to be run from /home/prash/share/analyses/Expipe/out |
| #fastqc already one for all samples. Pl check the folder out/ |
| ./bowtie2 -x ../../data/hg38/hg38 -1 ../../../../../admin1/share/km/raw_data/XGC-575-Z_R1.fq.gz -2 ../../../../../admin1/share/km/raw_data/XGC-575-Z71_R2.fq.gz > Z.sam |
| samtools import ../../data/hg38/hg38.fa Z.sam Z.bam |
| samtools sort Z.bam -o Z.sorted.bam |
| samtools index Z.sorted.bam Z.sorted.bam.bai |
| #samtools merge Z.merged.bam Z.sorted.* |
| samtools mpileup Z.sorted.bam -o Z.mpileup.bam |
| java -jar ../varscan/varscan.jar mpileup2snp Z.mpileup.sam > Z.mpileup.snps |
| java -jar ../varscan/varscan.jar mpileup2indel Z.mpileup.sam > Z.mpileup.indels |
| java -jar ../varscan/varscan.jar filter Z.mpileup.snps >Z.mpileup.snps.filter |
| java -jar ../varscan/varscan.jar readcounts Z.mpileup.sam >Z.mpileup.readcounts |
| samtools mpileup -uf ../../data/hg38/hg38.fa Z.sorted.bam \| bcftools view - > Z.var.raw.bcf |
| bcftools view Z.var.raw.bcf \| vcfutils.pl varFilter -D100 > Z.var.flt.vcf |
| samtools calmd -Abr Z.sorted.bam ../../data/hg38/hg38.fa > Z.baq.bam |
| samtools mpileup -uf ../../data/hg38/hg38.fa Z.baq.bam \| bcftools view - > Z.baq.var.raw.bcf |
| bcftools view Z.baq.var.raw.bcf \| vcfutils.pl varFilter -D100 > Z.baq.var.flt.vcf |
